# Adherence to precautions for preventing the transmission of microorganisms in primary health care: a qualitative study

**DOI:** 10.1186/s12912-017-0245-z

**Published:** 2017-09-11

**Authors:** Michely Aparecida Cardoso Maroldi, Adriana Maria da Silva Felix, Ana Angélica Lima Dias, Julia Yaeko Kawagoe, Maria Clara Padoveze, Sílvia Alice Ferreira, Sílvia Helena Zem-Mascarenhas, Stephen Timmons, Rosely Moralez Figueiredo

**Affiliations:** 1 Department of Nursing, Federal University of São Carlos, São Carlos, Brazil; 2 Education Institute, Hospital do Coração (HCor), São Paulo, Brazil; 3 Albert Einstein Instituto Israelita de Ensino e Pesquisa, São Paulo, Brazil; 4 Hospital Infection Division, São Paulo State Health Department, São Paulo, Brazil; 5 School of Nursing, University of São Paulo, São Paulo, Brazil; 6 Centre for Health Innovation, Leadership and Learning (CHILL), Nottingham University Business School, Nottingham, UK

**Keywords:** Infection prevention and control, Primary care, Standard precautions, Adherence, Qualitative study, Nursing, Focus group, Transmission

## Abstract

**Background:**

Health care associated infections (HAIs) are a source of concern worldwide. No health service in any country can be considered HAI risk-free. However, there is scarcity of data on the risks to which both patients and health workers are subject in non-hospital settings. The aim of this study was to identify issues that determine the adherence of professionals to precautions for preventing transmission of microorganisms in primary health care.

**Method:**

This was a qualitative study, using focus groups of primary health care staff, in two Brazilian municipalities. The data were analysed using content analysis.

**Results:**

Four focus groups were conducted with 20 professionals (11 community health workers, 5 nursing assistants and 4 nurses), and the analysed content was organized into four thematic categories. These categories are: *low risk perception*, *weaknesses in knowledge*, *insufficient in-service training* and *infrastructure limitations*.

Participants expressed their weaknesses in knowledge of standard and transmission based precautions, mainly for hand hygiene and tuberculosis. A lack of appropriate resources and standardization in sharps disposal management was also highlighted by the participants.

**Conclusion:**

The study points out the need to provide in-service training for professionals on the transmission of microorganisms in primary health care to ensure adequate level of risk perception and knowledge. Further recommendations include investment to improve infrastructure to facilitate adherence to precautions and to minimize the risk of disease transmission for both patients and health care workers.

## Background

Healthcare associated infections (HAIs) are a source of concern worldwide [1–3]. According to the World Health Organization [4] no health service in any country can be considered HAIs risk-free. However, there is scarcity of data on the risks to which both patients and health workers are subject in non-hospital settings. HAIs have mainly been considered to be “a hospital problem” and only a few studies have focused on the risk of acquiring infection due to procedures performed in primary health care (PHC) [5]. Procedures performed in PHC worldwide are mostly of low invasiveness. Due to this, the risk of acquiring HAIs in PHC has been considered to be less important [6–8]. Nonetheless, patients, healthcare professionals, family members, and caregivers may be at risk of acquiring infections as a result of exposure wherever health care is given. Hence, the lack of literature suggests this is an under-acknowledged problem.

Irrespective of the health care setting, infection prevention measures must be applied and are considered essential for quality care. Adherence to standard precautions (SP) and to transmission-based precautions (TBP) are among the core components for preventing the transmission of microorganisms [1, 9]. For instance, in low and middle income countries in which tuberculosis is highly prevalent, the risk of occupational acquisition is a real threat. This can jeopardize existing health human resources in places already suffering shortages of physicians and nurses.

Despite this, adherence to SPs by professionals in hospitals is less than optimal [10]. In PHC, little is known about actual adherence to precautions [6] and also other factors such as knowledge, attitudes, potential facilitators and barriers to achieving compliance. A recent study in 20 tuberculosis care facilities in Nigeria revealed flaws in the process and barriers that hindered the implementation of tuberculosis control measures [11]. Poor practice in tuberculosis prevention was also found in 52 primary health care settings in South Africa [12]. Diseases other than tuberculosis are also of worldwide concern due to their ability to affect both patients and healthcare workers. These include hepatitis, influenza, Ebola hemorrhagic fever, and coronavirus [13–17]. To our knowledge there is no study addressing PHC which seeks to understand the factors that contribute to compliance to SP and TBP. In this study, we aimed to explore knowledge and barriers to the implementation of precautions for the prevention of transmission of microorganisms in PHC, as well as those factors that determine the adherence of professionals to these measures.

## Method

### Study design

This is a qualitative study using focus groups [18] to obtain data. We chose a qualitative design to gather data from people in their natural work environment and allowing free interaction among participants, as we believed this would be the best approach to generate new information on this topic. We aimed to capture feelings, ideas, perceptions and attitudes of the participants in order to identify the knowledge PHC professionals had about SP and TBP, as well as issues that could affect adherence to such measures.

### Settings

The study was conducted in five PHC units, managed by the Brazilian Family Health Strategy (FHS) in two cities (population 220,000 and 15,000) in the state of São Paulo, Brazil. PHC is the gateway to the Brazilian public health system, which is based on universal coverage. PHC is provided in ambulatory units, which are healthcare settings not linked to hospitals. A particular feature of FHS is the role of Community Health Agents (CHA), lay members of the community who are paid to be part of the FHS team along with nursing assistants or technicians, registered nurses and doctors [19]. CHA usually has no previous professional training in health; they get only in-service training. They act as a communication link between users and the health service, identifying the needs of individuals and families enrolled in the FHS.

### Participants

Workers engaged in the FHS teams were invited to participate. As a qualitative study, a statistical sample size was not calculated, but 45 individuals were identified with relevant roles in the FHS in five PHC units. Professionals were recruited by direct invitation among those working in units suggested as study settings by nursing managers in the municipalities. Twenty professionals from different units agreed to participate and the final sample included 11 CHAs, 4 registered nurses, 5 nursing assistants or technicians. Four focus groups were conducted.

### Data collection

Focus groups took place during the working day and at the participants’ workplace from January to March 2014 and lasted on average 45 min 1 h (only one focus group lasted 25 min). The focus groups were run by two researchers who acted as the facilitator and the observer. To start and direct the conversation, a semi-structured script was used. We presented day-to-day situations, related to the risk of transmission of microorganisms, and encouraged the professionals to express their perceptions and opinions. Focus groups were conducted in Portuguese, and quotations have been translated into English for this report.

### Data analysis

Focus group interactions were audio recorded, transcribed *verbatim* and analyzed in depth. A content analysis technique was used to identify clusters of ideas and themes that emerged from the text. This technique has three main phases: preparation, organizing and reporting [20]. The statements were identified by professional category using an alphanumeric code to allow recognition of professional category and groups while maintaining anonymity of individuals. Nursing assistants and nursing technicians: “NA”; nurses: “N”; community health agent: “CHA” and 1, 2, 3, were used to differentiate individuals. The material shows the interpretations and experiences of these professionals in their daily work in the PHC, allowing us to understand behaviours relevant to SP and TBP.

### Ethics

The study was authorized by the Ethics Committee of the Federal University of São Carlos, note number 451,814. Participants received written and oral information regarding the research objectives and procedures and signed a written individual consent in accordance with Brazilian ethics regulation [21].

## Results

Four focus groups were conducted, with an average size of 5 professionals per group, totaling 20 professionals (11 CHAs, 5 nursing assistants and technicians, and 4 nurses, that agreed to participate). Participants were mostly women (*n* = 18), with an average of 4.9 years’ working experience in the PHC. Medical professionals were invited to participate in the focus groups, but none did.

From the analysis of the focus groups discussions, the ideas expressed were grouped in four thematic categories: *low risk perception*, *weaknesses in knowledge*, *insufficient in-service training* and *infrastructure limitations*.

### Category 1: Low risk perception

One of the main causes of the non-adoption of measures to prevent microorganism transmission in PHC was the perception by professionals that risks were low. The failure to recognise these risks suggests a failure to adopt the necessary precautions in their daily activities. The fact that professionals thought and said little about this theme suggests that it was not perceived as a risk. Risk perception was a motivator for decision making.

*“CHA 2: I’m kind of sloppy with it (precaution measures) (...) Just like now: before I was working all the time, I did not wash my hands and already [I] got a cookie (...).”*

*“N 1: No, we are not careful (...). But I think we talk too little about it, now that you are talking about it, I see it’s very important?!”*

Precaution measures were considered relevant during discussions in the focus group. On the other hand, adherence has not always been a priority, which can hinder its implementation in everyday life. PHC is not considered to be as risky as hospital environment for the acquisition of infections.

*“NA 2: Once I caught scabies taking care of someone who was homeless. So, these are things we do not really do on a daily basis, for lack of a proper work strategy. We work wrongly (...)”*

*“N 1: But in the PHC it is left on one side, we are more concerned about the hospital.”*

*“CHA 6: We know that is necessary, but in the moment, we do not even notice, it is by impulse, it is automatic.”*

*“N 3: (...) what is used is the same standard precautions such as aprons, gloves, sometimes a mask when bandaging, but otherwise there is no routine use. The use of precautions for contact, aerosols, I have not seen in PHC.”*

*“NA 3: (...) we are not doing the work correctly, for sure we are not, we are aware of that.”*

### Category 2: Weaknesses in knowledge

Although some participants showed some knowledge of SP and TBP, most did not usually apply it, due to difficulties in transferring knowledge into practice.

*“N 1: I think it is not very clear to the team, I think we need to talk more about it. I know the theory, but in the practice, I have doubts.”*

*“NA 3: What you are showing us here is something that we have to observe more and practice (...) no one gives any importance to this detail.”*

Focus group data showed some shortcomings in participants’ technical knowledge: these included hand hygiene, transmission of pulmonary tuberculosis and sharps disposal. Misconceptions expressed by participants about when and how to use alcohol hand rubs demonstrated insufficient knowledge of this subject. Some professionals did not recognize alcohol as a first option for hand hygiene. Others believed that an alcohol hand rub was not an alternative to hand washing but rather thought that they needed to use both hand washing and alcohol rubbing to achieve the best performance of hand hygiene. It is important to highlight there was no mention of knowledge of the recommendations of the “five moments of hand hygiene”.

*“N 4: At least they use alcohol after washing hands (...) because there’s no point using only alcohol gel and not hand washing. You have to do both.”*

*“NA 4: At the [PHC] Unit, I think, since we can wash our hands (...) there is not much need to use the alcohol gel (...). It’s optional, wanting to use alcohol gel after washing hands. But I think it’s more important handwashing than the use of alcohol gel.”*

*“NA 2: (...) hand washing with soap and water is what cleans and eliminates bacteria.”*

Doubts about the transmission of tuberculosis and protection measures were reported by the professionals. They were unsure about the indications for use of masks, in both PHC settings and home visits. In addition, they expressed uncertainty about how to advise patients about the use of masks when they go to the PHC unit for Directly Observed Therapy (DOT) of tuberculosis.

*“N 1: I have more questions concerning the use of mask at a home because in the house there may be a patient who refuses to accept a visit by the CHA. How will this be if the CHA uses a mask in the patient’s home? ”*

*“N 2: A patient’s resistance to come here to the unit with the mask is too much, especially in the early days. I do not know if it’s embarrassment or lack of guidance, but I see the refusal to wear the mask is extensive.”*

As for sharps disposal at home, participants showed flawed knowledge of the guidelines on segregation and final disposal of the waste generated.

*“N 3: The patient who makes use of insulin is told to throw the waste generated inside a plastic bottle, right?! Then he takes it to our Unit for us to discard it in the sharps box, not in the trash. ”*

*“N 2: They bring the plastic bottle firmly closed, it is rigid, right?! Then we give it to the nurse aide to put in the infectious waste.”*

### Category 3: Insufficient in-service training

The professionals recognized the need for discussion and training in-service regarding SP and TBP. It was clear that there had not been any training with the teams in this area. However, they considered that all healthcare professionals were supposed to be trained to recognize risks and respond appropriately.

*“N 1: We know little (...) I am a failure at this training.”*

*“NA 2: (...) in my case, I do what I have learned during the training course, but it was not someone here who trained us (...).”*

The provision of in-service training for CHAs on measures to prevent disease transmission was also considered relevant since they are not qualified professionals with formal healthcare training. CHAs should (at least) receive essential information regarding the modes of transmission of highly prevalent diseases and methods for self-protection and protection of patients.

*“CHA 7: During the visit, we can detect the [patient’s] symptoms [of some diseases], we make an appointment [for the patient] at the PHC unit, but [by that point] we have already had contact [with them] in the home. It is [only] in the unit that the person will be diagnosed with pulmonary tuberculosis. And [by that point] we had already visited and entered the home, and talked [with the patient] (...) ”.*

*“CHA 4: I think we, the CHAs, we must have training about pulmonary tuberculosis, and all health professionals need it.”*

The focus group participants mentioned that there is also insufficient training for housekeeping staff. Since housekeeping is provided through an outsourced private company, the development of in-service training is an even greater challenge.

*“NA 4: housekeeping teams are outsourced, and I believe that they have no guidance on how to clean the unit safely (...) She [housekeeping worker] was mopping (...) without using gloves, and [hand] wringing the mop out (...)”*

### Category 4: Infrastructure limitations

This category includes the opinions of professionals about the inadequacy of infrastructure regarding physical space, and deficits in the quality and availability of materials needed for good practice. These factors influenced the workers’ practice, hindering their daily work.

*“NA 5: So, there is no [appropriate] structure. I believe everyone is aware of what is wrong, and it’s all wrong. You are aware of it, but what can you do?! You try to do the best, but the environment does not help it.”*

*“N 2: (...) where the cleaning and disinfecting of equipment is done is [the same place] where we do [health care] procedures, it is where we perform specimen collection, it is where we do wound dressing, it is where we put the patient under observation (...) it is where intravenous medication is done, all that.”*

The professionals recognized that taking care of people who require TBP is hampered in many situations due to the lack of availability of appropriate rooms. Indeed, many PHC units in Brazil are not purpose-built but are residential houses adapted for health care.

*“NA 3: (…) there are patients under treatment for pulmonary tuberculosis who are [in the waiting room] near a child who is playing, and we do not know how to handle this situation.”*

*“N 1: (...) I do not separate children with chickenpox from others because there is no place here to separate [isolate] them.”*

Some participants thought that the materials provided were low quality and others that insufficient were provided.

*“N 4: (...) the gloves that come [from the manufacturer] are all pierced, most of them are pierced.”*

*“NA 4: No shortage of gloves, but [there is] no paper towels.”*

## Discussion

In recent decades, health care has changed from being predominantly hospital-based to being delivered in settings such as home care and ambulatory services. Therefore, risk assessment and implementation of good infection control practices need to be expanded beyond hospitals [7]. To the best of our knowledge, this is the first exploratory study, using a qualitative approach, to investigate infection prevention in PHC, bringing new insight about the subject and contributing to minimizing the global gap in this field.

### Low risk perception

The perception of risk directly influences the adherence of professionals to recommended measures [22]. Even in hospitals, although professionals know how to protect themselves from risks of injuries and infections, they do not always comply with safe practices [23]. The perception of risk and the adoption of biosafety measures constitute a challenge in PHC, and research in this area is scarce. Traditionally, the risks of HAIs in PHC had being considered low, but in a comprehensive literature review, no studies were found to provide epidemiological support for this claim [5].

The low perception of self-risk of infection was also discussed by a multinational study group highlighting the risk of extensively drug-resistant tuberculosis [24].

One study found an increased risk of *Mycobacterium tuberculosis* infection in health professionals, students, and CHAs, who are six times more likely to acquire the disease while caring for infected patients if they do not use specific protective measures [25].

The perception of low risk can be a major cause of shortcomings in adopting measures to prevent transmission of microorganisms.

### Weaknesses in knowledge

Our study demonstrated that health professionals in PHC during the focus groups had an initial perception of their own lack of awareness and knowledge on several issues in infection prevention. They said that they should think and talk more about the subject as a way to dissipate misconceptions and better translate knowledge into practice. Knowledge is known to be a first step for awareness of self-protection and patient protection [26]. Even fundamental knowledge on hand hygiene is far from being good in many outpatient settings [6, 10, 26]. Consequently, hand hygiene is less than optimal. For instance, a Brazilian study showed that hand hygiene was not performed by health professionals in approximately 60% of cases in a home care service. These professionals did not perform hand hygiene in 77% of instances when arriving at a patient’s home and in 38% when leaving them [6]. Another study demonstrated that in a PHC setting hand hygiene was rarely performed before care, ranging from 8% to 53.3% depending on the type of procedure; and after procedures such as capillary blood glucose monitoring only in 20% of instances. In intravenous medication administration, 53.3% washed hands prior to the procedure and 27.3% after that [27]. Nonetheless, this low compliance does not seem to be restricted to Brazil or indeed other low-middle income countries. A Spanish study found out that the adherence rate to hand hygiene was 8.1%, and that professionals washed their hands mainly after contact with the patient rather than before it [5].

### Insufficient in-service training

In the present study, when talking about the choice of products for hand hygiene, the professionals pointed to their beliefs of a higher efficacy of water and soap compared to alcohol. This is outdated information as since 2009 the World Health Organization has implemented a worldwide campaign recommending the use of alcohol hand rubs as the first option for hand hygiene [4]. The lack of current information reinforces that PHC is not receiving even minimal in-service education for infection prevention.

Practices need to be sustained by a good level of knowledge and scientific evidence, otherwise they may contribute to the spread of infections in the health care setting [28]. Furthermore, the lack of specific training for CHAs and housekeeping workers is of great concern. These professionals are even more vulnerable than health care professionals because they do not have formal education in health care and therefore, for them, in-service training is imperative [29, 30].

The subject of tuberculosis emerged strongly in the focus groups. Worldwide, patients harbouring *Mycobacterium tuberculosis* are being cared for in PHC; many of them while at the bacillary phase, mainly in countries with high prevalence [8, 31]. Therefore, it is essential that biosafety measures, such as the use of respiratory protection and cough etiquette, alongside environment control measures, as well as standards for triage and sputum collection from outpatients, are widely adopted in PHC [25, 31, 32]. The data obtained in our study indicated that professionals are unsure about these recommendations, probably due to insufficient in-service standards and training.

When performing home visiting, CHAs could have a key role in the early identification of individuals with respiratory symptoms. This could reduce the likelihood of those individuals attending PHC settings without precautions, thereby minimizing exposure of health professionals and other patients. However, a study demonstrated that CHAs were not able to recognise these symptoms [33]. They have potential extra exposure compared to other health professionals as they perform home visits more frequently. In addition, CHAs come from the same community as their patients, which implies they are experiencing similar social and economic determinants of health.

The stigma associated with tuberculosis may impair the adoption of some of these measures such as the use of respiratory protection (masks) [34]. However, the subject of stigma did not arise in the focus group but rather a lack of knowledge of guidelines. Once more, knowledge is key for awareness. This points to the need of systematic in-service training to minimize risk.

### Infrastructure limitations

Waste sharps may be generated due to health care procedures performed at home, particularly for diabetic patients. Nevertheless, professionals complained about a lack of straightforward recommendations on how to deal with them. Other researchers have pointed out the need for guidelines on appropriate disposal, segregation and transport of waste generated by health care provided in the patient’s home. Patients and families of those performing self-administration of injections should be guided about the management of sharps [6, 35]. The participants in the focus group expressed their concern about limitations of material resources, mainly the low quality of gloves provided at PHC. In addition, the PHC premises were unable to adequately accommodate patients with respiratory transmitted diseases such as tuberculosis and chickenpox. Performing good health practice requires the provision of appropriate infrastructure, personal protective equipment, environmental control and proper provision of equipment and supplies. The absence of these conditions affects the adequacy of work, resulting in low quality of care [36, 37].

Altogether, low risk perception, weaknesses in knowledge, insufficient in-service training and Infrastructure limitations show that HAIs prevention is far from being a priority at PHC. Perhaps, in countries where access to health care is very limited, concerns about the prevention of HAIs might be seen to be a luxury [38]. In most low-middle income countries efforts to provide universal health coverage are so challenging that prevention of infections due to health care associated infections might be seen as a secondary target. However, failures in preventing the transmission of microorganisms at PHC level can affect the entire health care system, and contribute to the spread of epidemiologically relevant pathogens. The major pandemics have shown that all health services must be prepared for an efficient and coordinated response to prevent amplification of any epidemic phenomena. This perspective was evident in episodes of Severe Acute Respiratory Syndrome - SARS, pandemic influenza and more recently in the epidemics related to Ebola virus [14, 39]. Nonetheless, the literature from high income countries is also quite silent about potential HAIs due to procedures in PHC, except for some coverage of outbreaks [9, 15–17].

We do not intend our data to be fully transferable worldwide. Nevertheless, the results points to the need for guidance, training and adequate provision of supplies and structure to promote compliance with essential measures to prevent HAI across the entire health care system.

## Limitations

This study did not aim to achieve saturation, but rather to explore the perceptions and opinions of professionals directly involved in the issue; therefore, sample and the focus group size was limited. Holding focus groups in PHC was difficult, since the clinical workload did not make it easy for professionals to participate. Further studies could consider the use of interviews as this might work better approach in PHC. Nevertheless, the number of people in the focus groups used in this study is broadly representative of the (small) PHC teams. There was a high degree of consistency in the data across the focus groups.

In this study, lack of participation by medical staff may have caused a tendency to focus on issues of interest to nursing professionals and CHAs. In addition to the very small teams, the engagement of physicians in group activities with other personnel is not possible in many situations. This is not unique to Brazil but also occurs in other contexts. Although infection prevention is a multidisciplinary effort, nurses and CHAs are a critical element in the health care team due not only their numbers but also due to their frequent, direct patient contact, and their role in education.

## Conclusion

This study identified, among PHC professionals, weaknesses of knowledge of SP and TBP, particularly in aspects related to timely and effective hand hygiene; adoption of protective measures before a (suspected or confirmed) case of pulmonary TB, and standardization of sharps disposal at home. Flaws in the infrastructure were pointed out as barriers to promoting adequate infection prevention measures. Undoubtedly, the first step in reaching awareness is promoting in-service training programs. By understanding professionals’ knowledge and their perceptions of barriers present in their daily work in PHC, we propose that the next step should be to design tailored interventions aiming at achieving improvements.

## Relevance to clinical practice

To ensure national preparedness to deal with epidemics and pandemics, all health care settings need to ensure a good level of adherence to infection prevention measures. Primary care is (worldwide) the first point of contact in dealing with infectious diseases. These findings show the main issues that should be addressed to improve infection control practice in primary care, to minimize the risk of disease transmission to both patients and health care workers.

Raising awareness by promoting knowledge is a key element of clinical practice. Notwithstanding this, to implement guidelines and in-service training, tailored to the local context, is very relevant to clinical practice since PHC is provided in such a variety of environments and situations.

## Abbreviations

CHA: Community Health Agents
FHS: Family Health Strategy
HAI: Healthcare associated infections
PHC: Primary Health Care
SP: Standard precautions
TB: Tuberculosis
TBP: Transmission-based precautions
